# Photocycloadditions and photorearrangements

**DOI:** 10.3762/bjoc.7.15

**Published:** 2011-01-26

**Authors:** Axel G Griesbeck

**Affiliations:** 1 University of Cologne, Department of Chemistry, Organic Chemistry, Greinstr. 4, D-50939 Köln, Germany; Fax: +49(221)470 5057

Organic synthesis using light as reagent and producing complex molecules from simple starting materials is realized in exemplary fashion in natural photosynthesis. Mankind still struggles for an efficient molecular system that mimics this natural process. In order to harvest light energy and to transform it into chemical energy, photochemical reactions must be studied and optimized for synthetic applications. Two major reaction paths that use electronic excitation are photocycloadditions and photochemical rearrangements. These reactions have been intensively investigated in recent decades in terms of regio-, stereo-, spin- and (electronic) configurational selectivities.

Prior to every photochemical reaction, an electronically excited state is generated either by direct light absorption or by energy transfer sensitization. This excited state has to be sufficiently well characterized in order to understand its chemical and physical behaviour. Organic molecules can react either as triplet or singlet excited states, often showing spin-specific reactivities and selectivities. Additionally, the nature of the excited state, as characterized by the state configuration, is crucial for the reactivity of the molecule, for example, nπ* states favour hydrogen abstraction and addition modes whereas ππ* states favour addition and electron transfer modes.

Photocycloaddition reactions can be performed in numerous ways using unsaturated substrates such as carbonyl compounds, Michael acceptors, monoalkenes, polyenes, or aromatic substrates leading to complex products that can be used in subsequent transformations. The carbonyl-ene photocycloaddition, for example, is an important route to oxetanes, products that have recently gained increasing attention as building blocks in organic synthesis as well as in materials science [1]. Photochemical rearrangements are impressive reactions with regard to the generation of complexity: 1,2- and 1,3-acyl shifts are known from carbonyl photochemistry, di-π-methane and oxa-di-π-methane rearrangements are processes that can occur with a remarkable increase in molecular and stereochemical complexity, as can *meta* arene photocycloadditions.

It was a great pleasure to act as the editor of this Thematic Series on photochemical reactions and I would like to thank all authors for their excellent contributions and the staff of the Beilstein-Institut for their support.

Axel G. Griesbeck

Cologne, January 2011
